# Characteristics of *Candida albicans* metabolism of glucose and two sugar substitutes, xylose and xylitol and effect of these substitutes on glucose metabolism from a cariogenic perspective

**DOI:** 10.1080/20002297.2026.2626130

**Published:** 2026-02-07

**Authors:** H. R. F. Mousa, Y. Abiko, J. Washio, S. Sato, N. Takahashi

**Affiliations:** a Division of Oral Ecology and Biochemistry, Tohoku University Graduate School of Dentistry, Sendai, Japan; b Pediatric Dentistry and Dental Public Health Department, Faculty of Dentistry, Ain Shams University, Cairo, Egypt; c Tohoku University Graduate School of Dentistry, Sendai, Japan

**Keywords:** Xylose, xylitol, glucose, *Candida albicans*, acidogenicity, dental caries

## Abstract

**Objectives:**

Carbohydrate metabolism and subsequent acid production of *Candida albicans* remain insufficiently understood. *C. albicans* may utilize xylitol, but its cariogenic implications are understudied. This study examined growth and metabolism of glucose, xylitol and precursor xylose by *C. albicans* and their effects on glucose metabolism.

**Methods:**

*C. albicans* JCM1537 was cultured in YNB medium containing 1% glucose, xylose, xylitol or xylose- or xylitol-glucose combinations. Acid production from cells grown on each substrate was assessed by pH-stat system at pH 7.0 using 10 mM substrates. Metabolic end-products were quantified by HPLC and enzymatic methods. Carbon recovery and redox balance of glucose metabolism were calculated stoichiometrically.

**Results:**

Growth and acid production on xylose or xylitol were much lower than on glucose, with no inhibitory effect of xylose or xylitol observed. Glucose carbon was distributed as 50.48% ethanol, 21.95–24.72% bicarbonate, 5.70% glycerol, 2.88% organic acids and 0.12% acetaldehyde, yielding 81.07–84.48% recovery and 93.26–93.97% reduction–oxidation balance.

**Conclusions:**

Although xylose and xylitol did not inhibit *C. albicans* glucose metabolism, their limited growth and acidogenicity suggest low cariogenic potential. The overall view of glucose metabolism, including high ethanol production, provides new insights into the metabolic impact of *C. albicans* within the oral microbiome.

## KEY MESSAGES

- *Candida* *albicans* was able to metabolize xylitol and its precursor xylose; however, rates of growth and acid production were lower than on glucose.
- Xylose and xylitol did not inhibit glucose metabolism in *Candida* *albicans.*
- Under aerobic conditions, and with an abundance of glucose, *Candida* *albicans* converts most of the consumed glucose into ethanol and carbon dioxide, and some into organic acids.

## Introduction

*Candida albicans* is the most isolated yeast in the oral cavity, widely believed to be a commensal microorganism that causes no harm to healthy individuals under normal circumstances. However, multiple studies have shown that it is associated with dental caries, especially with early childhood caries (ECC) [1], and potentially forms a synergistic relationship with *Streptococcus mutans*, leading to thicker biofilms and enhanced cariogenicity [2]. Our previous study [3] also showed that under both aerobic and anaerobic conditions, *Candida* species has its own high-level acidogenic potential that may contribute to the demineralisation of hard dental tissues, and it is markedly resistant to fluoride at regular toothpaste concentrations (up to 1,500 ppm). These findings suggest that the involvement of *Candida* species in dental caries may have been underestimated, and that in addition to supporting *S. mutans'* colonisation and enhancing its resistance to antimicrobial agents [4], it may also have other unknown functions based on its biological characteristics. However, the basic biochemistry of *Candida* species, such as sugar metabolism and resulting acid production, remains poorly understood. Less than 10% of the acids produced from sugars have been identified [3,5], and the fate of ingested sugars, i.e. how they are metabolised and what end products are produced, is still unknown.

Xylitol and its precursor xylose are two common naturally occurring sugar substitutes that have seen an increase in use in recent years. Despite being less sweet than sucrose [6], their lower caloric intake, lower glycemic index and insulin-independent metabolic pathway made them rise in popularity as weight loss or diabetes-friendly sweeteners [7,8]. In the oral cavity, xylitol is not fermented by oral bacteria and therefore does not cause caries, and it also inhibits growth, biofilm formation and acid production of *S. mutans* [9].

One of the possible reasons for the inhibition of *S. mutans* growth and acid production is considered to be that xylitol competes with other carbohydrates for uptake and is then metabolised, resulting in a metabolically futile cycle. When xylitol is taken up into cells, it is phosphorylated to xylitol-5-phosphate via the phosphoenolpyruvate-dependent phosphotransferase system (PEP-PTS). However, since xylitol-5-phosphate is not further metabolised, it accumulates in the cells, is converted back to xylitol by releasing phosphorylation energy, and is then excreted from the cells. This process results in a waste of energy [10]. However, this effect varies depending on the uptake priority of other carbohydrates; glucose showed the highest inhibition, while fructose exhibited almost no inhibition [11]. This is considered to be because a fructose-specific PEP-PTS is used for xylitol uptake, and when fructose coexists, fructose is taken up more preferentially than xylitol [10]. In addition, it should be noted that *S. mutans* may acquire xylitol resistance through long-term exposure to xylitol [12]. Aside from *S. mutans*, the effect of xylitol (and xylose) on other oral microbes remains unclear, although caries is a complex disease resulting from an imbalance in the interactions among multiple species within the oral microbiome, due to predisposing environmental conditions, not simply caused by *S. mutans* [13,14].

Previous studies showed that xylitol may inhibit biofilm formation of *C. albicans* [15], or decrease its numbers [16]; however, the effect on acid production has not been investigated. Nevertheless, unlike *S. mutans*, some yeasts are known to possess the ability to ferment xylose and xylitol strictly under aerobic conditions and utilise them as an energy source through the pentose phosphate pathway [17], albeit some, including *C. albicans*, still show a preference for glucose [18]. Despite this, the effect of yeast metabolism of xylitol and xylose on oral health has not been sufficiently investigated, and neither has the acidogenicity nor cariogenicity of the resulting metabolic end products of these pathways compared with glucose.

Therefore, in this study, we examined glucose metabolism of *C. albicans* and assessed whether xylitol or xylose exerts inhibitory effects when combined with glucose, as well as whether *C. albicans* can grow solely on xylitol and xylose. We further compared the acid production of *C. albicans* from xylose, xylitol and glucose, when grown solely on each of these substrates. In addition, we quantified non-acidic end products, including ethanol, glycerol and acetaldehyde, as well as acidic end products, and compared these products with respect to the amount of glucose utilised, aiming to gain a deeper understanding of the stoichiometry of glucose metabolism in *C. albicans*.

## Materials and methods

### *Candida* strains and growth conditions

*C. albicans* JCM1537^T^ was provided by RIKEN BRC through the National Bio-Resource Project of MEXT, Japan and was used for all experiments. *C. albicans* was grown on tryptone yeast glucose (TYG) agar plates, containing 1.7% tryptone (Becton Dickinson, Franklin Lakes, NJ, USA), 0.3% yeast extract (Becton Dickinson), 0.5% NaCl, 50 mM potassium phosphate buffer solution (PPB, pH 7.0) and 1% glucose (FUJIFILM Wako Pure Chemical Corporation, Osaka, Japan) at 37 °C for 24 h under aerobic conditions and then maintained refrigerated at 4 °C. Two other clinical isolates (JCM2903 and JCM2085, provided by RIKEN BRC through the National Bio-Resource Project of MEXT, Japan) were used for preliminary growth and pH-stat experiments to confirm the consistency of results, and to confirm the ability of different *Candida* strains to grow on xylose/xylitol (growth data of all three strains is shown in Figure S2).

Yeast Nitrogen Base (YNB) medium without amino acids (Thermo Fisher Scientific K.K., Tokyo, Japan) was used as a minimal medium for growth and pH-stat experiments. *Candida* cells were grown (pH 5.0, 37 °C) on YNB medium supplemented with one of these sweeteners (glucose/xylose/xylitol, FUJIFILM Wako Pure Chemical Corporation, Osaka, Japan) as a sole energy source, or cells were alternatively grown on glucose used in combination with either xylose or xylitol to test if there was an inhibitory effect.

### Culture growth in minimal medium supplemented with xylose and xylitol

*C. albicans* was grown at 37 °C for 24 h on TYG agar plates. Then, colonies of *Candida* species were collected and suspended in YNB medium and adjusted to (OD_520 nm_ = 1.0), 1 mL of this suspension was added to each test tube, and it was mixed with ten millilitres of YNB medium (supplemented with 1% w/v glucose, 1% w/v glucose plus 1% w/v xylose, 1% w/v glucose plus 1% w/v xylitol, 1% w/v xylose, 1% w/v xylitol or no carbon source at all [3]). The cultures were incubated at 37 °C under static, aerobic conditions. OD at 520 nm was measured throughout the growth process.

### Measurement of proton production activity

*Candida* cells were precultured at 37 °C for 24 h with shaking at 225 rpm [19] to minimise growth time and maintain metabolic activity in YNB medium supplemented with either glucose, xylose or xylitol as described above and then transferred to fresh YNB medium supplemented with the same sweetener. The cells were harvested in the logarithmic growth phase (OD_520 nm_ = 0.8–1) by centrifugation (15,000 × g, 7 min, 4 °C). Then, they were washed three times using washing buffer (2 mM PPB containing 150 mM KCl and 5 mM MgCl_2_, pH 7.0). The cell pellets were resuspended in the same buffer and stored on ice until use.

A pH-stat system (aerobic environment: AUTO pH-stat, model AUT-501; Toa Electronics, Tokyo, Japan) was used to monitor and quantify proton production at pH 7.0, as described previously [3]. Before each usage, the pH-stat system was calibrated using standard pH solutions (pH 6.86 and 4.01, FUJIFILM Wako Pure Chemical Corporation, Osaka, Japan). The cell suspensions were prepared using wash buffer (OD_520 nm_ = 3.46 ± 0.1), and the number of cells was measured (7.13 × 10^7^ ± 7.76 × 10^6^).

The suspensions were preincubated at 37 °C for 3 min and wash buffer (or another sweetener in the case of combination with glucose to assess the inhibitory effect) was added, and then they were incubated for a further 4 min. Test sweeteners were then added to the reaction mixture at a final concentration of 10 mM and proton production was monitored for 30 min. Upon the decrease of the reaction mixture pH due to proton production by the cells, an appropriate amount of 60 mM KOH was automatically dispensed to maintain a constant pH at 7.0, and proton production was then estimated from the amount of KOH consumption, as explained previously [20].

During the pH-stat assay, 0.9 mL of reaction mixture was collected just before and 30 min after addition of the substrate. Then, 0.1 mL of 6 *N* perchloric acid were added to each sample to stop further metabolism [21]. The samples were frozen at −30 °C and later used for analysis of acidic and non-acidic end products.

### Analysis of acidic end products

The samples collected during the pH-stat experiments were thawed and filtered through a polypropylene membrane (pore size: 0.20 μm; Toyo Roshi Ltd., Tokyo, Japan). The filtrates were analysed by high-performance liquid chromatography (HPLC; Shimadzu Prominence LC-20AD, Shimadzu Co. Ltd., Kyoto, Japan) and Shimadzu LabSolutions software V5.42 SP3. HPLC analysis conditions were as follows: Separation was performed by ion-exclusion using two Shim-pack SCR-102H columns (300 mm × 8.0 mm *i.d*.) connected in series (Shimadzu Co., Ltd.). The mobile phase consisted of a 5 mmol/L aqueous solution of *p*-toluenesulfonic acid (Shimadzu Co., Ltd.), with a flow rate of 0.8 mL/min and a total run time of 40 min. Detection was performed using a post-column pH-buffered electroconductivity detection system with a CDD-6A detector (Shimadzu Co., Ltd.).

Target acidic end products included succinate, citrate, pyruvate, malate, fumarate, lactate, formate and acetate [22,23]. *α*-ketoglutarate was excluded from the analysis because it was present at a constant level in the candidal suspension. The unknown peak, presumed to be carbonic acid, was also excluded due to its volatility and the difficulty of accurate quantification (Figure S1). It was confirmed that no product peak was detected after 40 min. The concentrations of organic acids were automatically recorded and calculated by comparing with the values of the standard solutions (Shimadzu LabSolutions software V5.42 SP3, Shimadzu Co. Ltd.). The standard solutions were dissolved into a mixed sample containing 1 mM of each target acidic product, according to the manufacturer's instructions and a single-point calibration was performed before each usage. Measurements of all target acidic end products in the samples were within the recommended range for linearity according to the manufacturer's instructions. Representative chromatograms of the sample and standard solutions are shown in Figure S1.

### Analysis of non-acidic end products and glucose

The collected reaction mixture during pH-stat experiments of the control and glucose samples (grown on YNB-glucose) were thawed and used for analysis of glycerol, ethanol and acetaldehyde, just before and 30 min after the addition of buffer/glucose. Additionally, measurement of residual glucose was conducted just before adding 10 mM glucose, and once more after 30 min of the reaction to measure the initial and unused remaining amounts in each sample.

Glycerol, ethanol, acetaldehyde and glucose were quantified using Enzytec™ Liquid Glycerol E8360, Enzytec™ Liquid Ethanol E8340, Enzytec™ Liquid Acetaldehyde E8300 and Enzytec™ Liquid D-Glucose E8140, respectively (J.K. International, Tokyo, Japan). All aforementioned quantitative assays were performed by enzymatic methods coupled with quantifying the conversion of NAD^+^ to NADH at 340 nm using a spectrophotometer (UV-1800, Shimadzu Co., Ltd., Kyoto, Japan).

### Stoichiometric analysis

To stoichiometrically analyse glucose metabolism of *C. albicans*, the carbon recovery rate was calculated from the amounts of all acidic and non-acidic end products obtained in the present study and the amount of glucose consumed during the process by the following formula:

(Sumofnumberofμmolesofcarbonofallacidicandnon-acidicend-productsfor30minutes)/(numberofμmolesofcarbonatomsofconsumedamountofglucosefor30minutes)x100%

To determine the number of μmoles of carbon generated, carbon numbers of formate, acetate, pyruvate, malate, fumarate, succinate, bicarbonate, ethanol, acetaldehyde, glycerol and glucose were set at 1, 2, 3, 4, 4, 4, 1, 2, 2, 3 and 6, respectively. Among the protons measured by the pH-stat system, the excess quantity that exceeded the corresponding amount of organic acids identified by HPLC were assumed to be CO_2_ (resulting from metabolic processes) that dissolved in water and became bicarbonate ions (CO_2_ + H_2_O → H_2_CO_3_ → H^+^ + HCO_3_^-^). The number of μmoles of carbon atoms was then calculated from the amount of bicarbonate. Adjustments were made to all calculations for dilution due to the addition of perchloric acid, changes in the final volume throughout the experiments, and number of cells in each sample. Additionally, the Henderson-Hasselbalch equation was applied to calculate the dissociation of bicarbonate using pKa of 6.35 at pH 7.0 to correct the number of μmoles of bicarbonate. Organic acids detected by HPLC have pKa values sufficiently below 7.0 and were therefore considered to have 100% dissociated and released protons.

Furthermore, the amount of reducing power (2 H) and CO_2_ produced or consumed during glucose metabolism was estimated based on the theoretically expected amounts resulting from the production of measured acidic and non-acidic metabolites following two expected metabolic pathways. One was based on glycolysis, ethanol production, acetate production and glycerol production (excluding TCA cycle), while the other included the reverse TCA cycle (phosphoenolpyruvate + CO_2_ → oxaloacetate; oxaloacetate + 2 H → malate; malate → fumarate + H_2_O; fumarate + 2 H → succinate) in addition to all previously mentioned pathways. The reduction–oxidation balance for both models was calculated using the following formula:

[(Totalamountof2Hproduction)/(totalamountof2Hconsumption)]x100%

In addition, net CO_2_ production was calculated from the predicted CO_2_ production and consumption based on the metabolic pathways used to calculate the reduction‒oxidation balance. The calculations were performed twice separately: initially assuming that the TCA cycle was not involved, and once again considering that the reverse TCA cycle was involved. Net CO_2_ production was also used to calculate the carbon recovery rate, as was CO_2_ production calculated from proton production undetectable by HPLC, as described above.

### Statistical analysis

Growth data were logarithmically transformed and statistically analysed using the Tukey (HSD) test. Dunnett's test was used to compare the effect of xylose/xylitol on proton production from glucose to the glucose-only control (assessed by pH-stat system), and compare proton production from xylose/xylitol-only to a negative control. Multiple comparison tests were not preceded by an omnibus statistical test. JMP Student Edition 18 software was used for statistical analyses, and *p*-values < 0.05 were considered significant.

## Results

### Growth of *C. albicans* in the presence of different sweeteners

**Figure 1.**
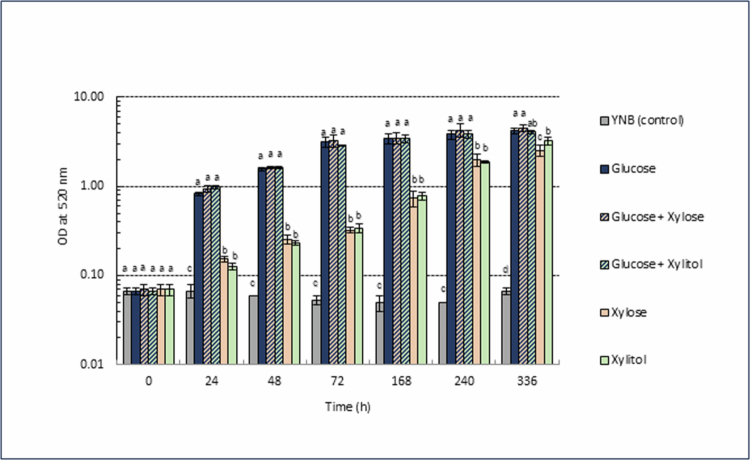
Growth (OD_520_) of *C. albicans* and the effect of xylose/xylitol under aerobic conditions. Data are shown as the mean ± standard deviation (SD) of 3 independent experiments. Tukey (HSD) test, bars not sharing any letters at each time-point are statistically significant.

*C. albicans* was able to grow on all three sweeteners separately (glucose, xylose and xylitol), under aerobic conditions (Figure 1), but growth on xylose and xylitol was significantly lower than that on glucose until the 240-h time-point; their growth rates had almost caught up by 336 h, although both remained significantly lower. Neither xylitol nor xylose had an inhibitory effect on growth when present simultaneously with glucose, and differences were non-significant compared with glucose at all time-points (Figure 1).

### Proton production by *C. albicans*

**Figure 2.**
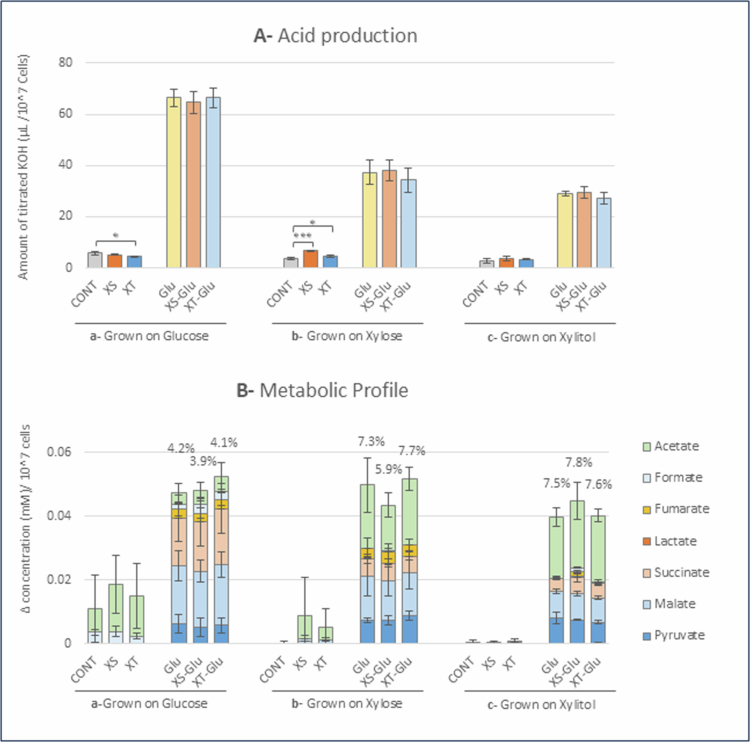
(A) Acid production (amount of 60 mM KOH consumed) resulting from metabolism of different sweeteners by *C. albicans* grown on: (a) YNB medium supplemented with 1% glucose, (b) YNB medium supplemented with 1% xylose, (c) YNB medium supplemented with 1% xylitol. Data are shown as the mean ± standard deviation (SD) of 3 independent experiments. Dunnett's test. ***p < 0.001, *p < 0.05. Cont, XS, XT, Glu, XS-Glu and XT-Glu represent the following substrates: nothing, xylose, xylitol, glucose, both xylose and glucose and both xylitol and glucose, respectively. (B) Metabolic profile resulting from metabolism of different sweeteners by *C. albicans* grown on: (a) YNB medium supplemented with 1% glucose, (b) YNB medium supplemented with 1% xylose, (c) YNB medium supplemented with 1% xylitol. Data are shown as the mean ± standard deviation (SD) of 3 independent experiments. Numbers on the top of glucose utilisation columns indicate the ratio of identified metabolites through HPLC analysis to total acid production from glucose. Cont, XS, XT, Glu, XS-Glu and XT-Glu represent the following substrates: nothing, xylose, xylitol, glucose, both xylose and glucose and both xylitol and glucose, respectively.

Cells of *C. albicans* grown on glucose produced most protons from glucose, compared with those grown on xylose or xylitol (Figure 2A). Proton production from glucose by *C. albicans* cells grown on glucose, xylose or xylitol was not inhibited by xylose or xylitol (Figure 2A).

The cells of *C. albicans* grown on glucose did not produce more protons from xylitol or xylose than the control (no substrate) (Figure 2A-a), indicating no proton production from xylose or xylitol by glucose-grown cells. In addition, proton production from xylitol was significantly lower compared with the control, while that from xylose was non-significantly lower than the control.

On the other hand, cells of *C. albicans* grown on xylose or xylitol exhibited higher proton production than controls. Cells grown on xylose (Figure 2A-b) were able to produce significantly more protons from xylose (*p* < 0.001), followed by xylitol (*p* < 0.05), while those grown on xylitol (Figure 2A-c) produced a very small amount of protons from both substrates, being higher than the control but not significant.

### Acidic end products produced by *C. albicans*

Cells of *C. albicans* grown on glucose produced malate and succinate mainly from glucose (Figure 2B-a). Combining glucose with xylose or xylitol did not change the metabolic profile. The ratio of the total amount of organic acids to protons produced from glucose metabolism was only 3.9–4.2%. The cells appeared to produce acids such as acetate from xylose and xylitol (Figure 2B-a); however, as described previously, the amount of protons produced was smaller than that of the control. Therefore, it is unreasonable to consider these as metabolic end products from xylose or xylitol.

Contrastingly, cells grown on xylose (Figure 2B-b) or xylitol (Figure 2B-c) clearly produced larger amounts of acetate from glucose, and lower rates of malate, succinate and formate compared with those grown on glucose. The cells grown on xylose produced small amounts of acidic end products from xylose and xylitol, with acetate being the main product (Figure 2B-b); however, the cells grown on xylitol did not produce acidic end products from xylose or xylitol (Figure 2B-c).

### Total identified end products of glucose metabolism

**Figure 3.**
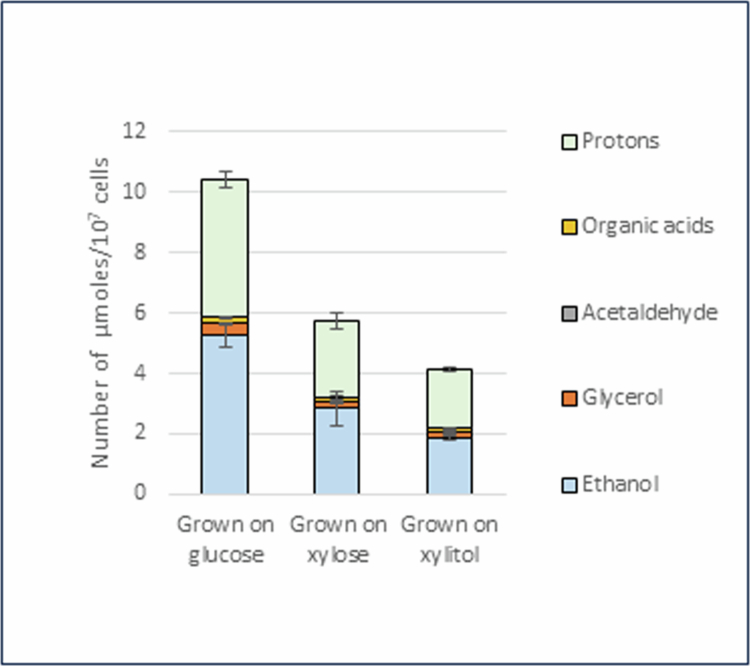
Total identified end products of glucose of *C. albicans* grown on YNB medium supplemented with 1% glucose, 1% xylose or 1% xylitol. Data are shown as the mean ± standard deviation (SD) of 3 independent experiments.

Surprisingly, cells grown on glucose produced the most protons (considered to be bicarbonate) and non-acidic end products, with ethanol being the most abundant of the latter (Figure 3). Organic acids (total of formate, acetate, pyruvate, lactate, malate, fumarate and succinate) accounted for only a small proportion of the end products, as did acetaldehyde. Cells grown on xylose and xylitol also produced a similar profile of end products from glucose, but in lower amounts than cells grown on glucose (Figure 3).

### Stoichiometric analysis of glucose metabolism by cells of *C. albicans*

Cells of *C. albicans* grown on glucose produced glycerol, ethanol and acetaldehyde from glucose, in addition to acidic end products, including formate, acetate, pyruvate, malate, fumarate, succinate and bicarbonate (Figures 2 and 3). These were summarised along with the amount of glucose consumed during 30 min of metabolism, to deduce the amounts of carbon consumed as glucose and amounts of carbon produced as metabolic end products (Table 1, upper panel). The amount of carbon derived from bicarbonate was calculated using three values, as described in Materials and Methods: the amount of protons exceeding the quantity of organic acids detected by HPLC, net CO_2_ produced by hypothesised metabolic pathways (without involvement of the TCA cycle), and net CO_2_ produced by hypothesised metabolic pathways (with involvement of the reverse TCA cycle) (Table 1, upper panel). Based on stoichiometric calculations, the carbon recovery rate was calculated to be 81.07–84.48% (Table 1, lower panel), and it was estimated that 50.48% of the carbon content was converted to ethanol, 21.95–24.72% to bicarbonate, 5.70% to glycerol, 2.88% to organic acids and 0.12% to acetaldehyde (Table 1, shown in parentheses in the second column of the upper panel).

**Table 1.** Carbon recovery and reduction–oxidation balance based on stoichiometric calculation of glucose metabolism by *C. albicans*.

				µmoles of 2 H produced/10^7^ cells^c^	µmoles of CO_2_ produced/10^7^ cells^c^
	µmoles/10^7^ cells	µmoles of carbon/10^7^ cells	(%)^g^	µmoles of 2 H consumed/10^7^ cells^c^	µmoles of CO_2_ consumed/10^7^ cells^c^
Acidic end product					
Formate	0.01 ± 0.00	0.01 ± 0.00	(0.03)	-	-
				0.01 ± 0.00	0.01 ± 0.00
Acetate	0.01 ± 0.01	0.02 ± 0.02	(0.11)	0.02 ± 0.02	0.02 ± 0.02
				-	-
Pyruvate^a^	0.02 ± 0.01	0.07 ± 0.03	(0.31)	0.02 ± 0.01	-
				-	-
Malate	0.06 ± 0.01	0.26 ± 0.06	(1.23)	0.06 ± 0.01^b^	
				0.06 ± 0.01^b^	0.06 ± 0.01^b^
Fumarate	0.01 ± 0.01	0.04 ± 0.03	(0.20)	0.01 ± 0.01^b^	
				0.01 ± 0.01^b^	0.01 ± 0.01^b^
Succinate	0.05 ± 0.02	0.21 ± 0.10	(1.00)	0.05 ± 0.02 ^b^	
				0.10 ± 0.04 ^b^	0.05 ± 0.02^b^
Bicarbonate	4.59 ± 0.28^d^	4.59 ± 0.28^d^	(21.95)^d^		
	5.30 ± 0.43^e^	5.30 ± 0.43^e^	(25.33)^e^		
	5.17 ± 0.45^b^	5.17 ± 0.45^b^	(24.72)^b^		
Non-acidic end product					
Ethanol	5.28 ± 0.41	10.56 ± 0.82	(50.48)	5.28 ± 0.41	5.28 ± 0.41
				5.28 ± 0.41	-
Glycerol	0.40 ± 0.03	1.19 ± 0.10	(5.70)	-	-
				0.40 ± 0.03	-
Acetaldehyde	0.01 ± 0.01	0.02 ± 0.01	(0.12)	0.02 ± 0.01	0.01 ± 0.01
				0.01 ± 0.01	-
Glucose consumed	3.49 ± 0.17	20.92 ± 1.01	(100)		
Carbon produced (µmoles/10^7^ cells)		estimated including excess protons compared to organic acids detected by HPLC			16.96
		estimated including CO_2_ from metabolic pathways except TCA cycle			17.67
		estimated including CO_2_ from metabolic pathways including reverse TCA cycle			17.55
Carbon consumed (µmoles/10^7^ cells)		calculated from glucose consumed			20.92
Carbon recovery^f^		carbon produced estimated including excess protons compared to organic acids detected by HPLC			*81.07* ^d^
		carbon produced estimated including CO_2_ from metabolic pathways except for TCA cycle			*84.48* ^e^
		carbon produced estimated including CO_2_ from metabolic pathways including the reverse TCA cycle			*83.88* ^^b^^
2 H produced (µmoles/10^7^ cells)		estimated from metabolic pathways except TCA cycle			5.35
		estimated from metabolic pathways including reverse TCA cycle			5.48
2 H consumed (µmoles/10^7^ cells)		estimated from metabolic pathways except TCA cycle			5.69
		estimated from metabolic pathways including reverse TCA cycle			5.87
Reduction–oxidation balance		calculated from metabolic pathways except TCA cycle			*93.97*
		calculated from metabolic pathways including reverse TCA cycle			*93.26*

a Calculated as pyruvate in this study.

b Calculated as bicarbonate from CO2 estimated from metabolic pathways including reverse TCA cycle.

- No production/consumption estimated.

c Estimated from pathways involving production/consumption of 2H/CO2.

d Calculated as bicarbonate from excess protons compared to organic acids detected by HPLC.

e Calculated as bicarbonate from CO2 estimated from metabolic pathways except TCA cycle.

f [(Total carbon recovered as metabolic products) / (Total carbon derived from consumed glucose)] × 100%.

g Percentage of carbon recovered as metabolic products, assuming the carbon content of consumed glucose is 100%.

Furthermore, the reduction–oxidation balance calculated from the production and consumption of reducing power (2 H) estimated from the assumed metabolic pathways (Table 1, upper panel) was 93.97 and 93.26% when estimated from the metabolic pathway excluding the TCA cycle and when estimated from the metabolic pathway including the reverse TCA cycle, respectively (Table 1, lower panel).

## Discussion

### Growth of *C. albicans* on glucose, xylose, xylitol and their combination with glucose

**Figure 4.**
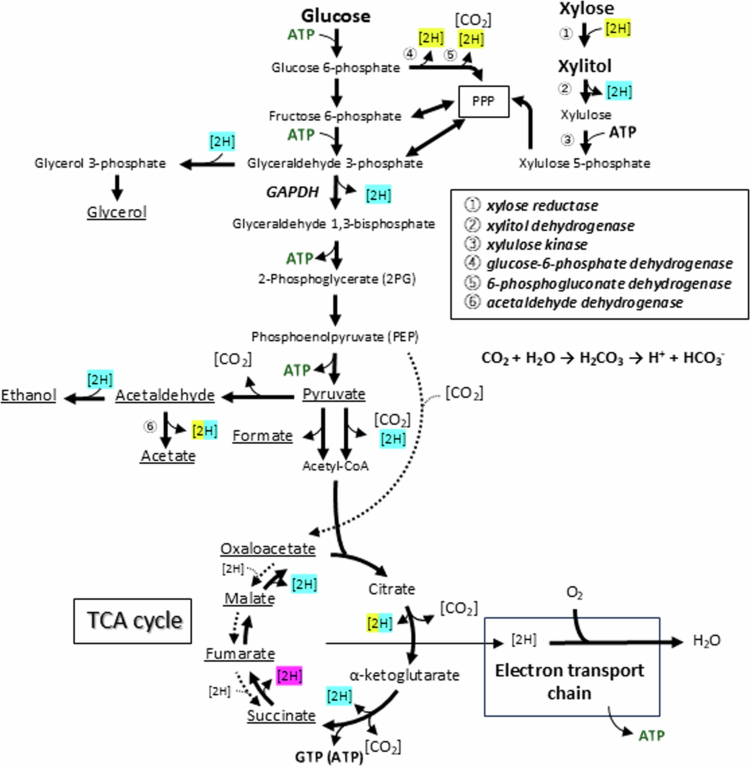
Proposed pathways for glucose, xylose and xylitol metabolism and reduction–oxidation balance maintenance in *C. albicans*. Yellow highlights indicate NADP^+^/NADPH-dependent reactions, blue highlights indicate NAD^+^/NADH-dependent reactions and the purple highlight indicates an FAD/FADH_2_-dependent reaction. The reactions highlighted in both yellow and blue are NAD(*P*)^+^/NAD(*P*)H-dependent reactions. PPP stands for the pentose phosphate pathway, and circled numbers indicate required metabolic enzymes for the corresponding reactions. The dashed arrows indicate the metabolic pathway of the reverse TCA cycle predicted in the present study.

In this study, we investigated the growth of *C. albicans* on xylitol, and its precursor xylose, compared with glucose. We found that unlike traditional cariogenic bacterial species such as *S. mutans*, *C. albicans* was able to survive on xylose and xylitol solely and even grow slowly under aerobic conditions (Figure 1). It is well-known that xylitol is not metabolised by oral bacteria, and furthermore, *S. mutans* undergoes a metabolically futile cycle: xylitol is taken up to produce xylitol-5-phosphate that is then not metabolised further and is expelled from the cell, wasting the high energy phosphoryl bond [10]. This cycle causes metabolic and growth inhibition in *S. mutans*, primarily on glucose. However, many yeasts are known to be able to metabolise xylose and xylitol into xylulose-5-phosphate, and further metabolise it through the pentose phosphate pathway as an energy source when needed (Figure 4) [17]. The present study revealed that *C. albicans* shared this metabolic property. Nevertheless, under the experimental conditions, growth on xylose and xylitol was markedly lower compared with that on glucose, suggesting that xylose and xylitol are also less cariogenic substrates for *C. albicans* species.

The slow growth and low acid production on xylose and xylitol likely reflects the accumulation of intracellular NADH due to limited oxygen availability, slowing overall metabolism and reducing ATP production. Yeasts are known to convert xylose to xylitol with NADPH consumption, and further to xylulose with NADH production (Figure 4, enzymes ① and ②), in which NADH must be recycled to NAD^+^ by oxidation using oxygen such as oxygen-linked NADH oxidase for smooth metabolism. Yeast cells can re-oxidise NADH to NAD^+^ through glycerol and ethanol production as redox sinks (Figure 4) [24]; however, this mechanism is insufficient in the case of xylose and xylitol metabolism due to the extra molecule of NADH produced compared with glucose. Therefore, metabolism of pentoses in the absence of oxygen is theoretically impossible for most yeasts, and the observation that no such yeast exists in nature [17] supports this theory. Although forced oxygenation, such as shaking culture, is expected to improve growth on xylose and xylitol, it is highly unlikely that a similar environment exists in the oral cavity. Therefore, our findings suggest that in deeper layers of plaque, where oxygen concentrations are low, growth and acid production resulting from xylose or xylitol metabolism by *Candida* cells is minimal.

Another reason for the slow growth and acid production of *C. albicans* on xylose could be that NADPH is required but not produced via xylose metabolism. While the reduction of xylose to xylitol is catalysed by NADPH-dependent xylose reductase (Figure 4, enzyme ①), many yeasts, including *C. albicans*, are considered to lack a transhydrogenase that facilitates the conversion between NADH and NADPH. Furthermore, the source of NADPH is generally limited to NADPH-generating enzymes (Figure 4, enzymes that produce reducing power [2 H], shown in yellow), glucose-6-phosphate dehydrogenase (Figure 4, enzyme ④), 6-phosphogluconate dehydrogenase (Figure 4, enzyme ⑤), aldehyde dehydrogenase and isocitrate dehydrogenase (Minard and McAlister-Henn, 2005). In the presence of glucose, *C. albicans* can generate NADPH mainly via glucose-6-phosphate dehydrogenase and 6-phosphogluconate dehydrogenase, but this is not feasible when pentoses are the sole energy sources available (Figure 4). Growth and acid production on xylose, albeit slowly, suggests that NADPH supply may occur slowly through other metabolic enzymes, such as NAD(*P*)-dependent acetaldehyde dehydrogenase [25–27] (Figure 4, enzyme ⑥), as discussed below.

Apart from oxygen availability, other factors such as the uptake mechanism might be partially responsible for the slow growth and acid production on xylose and xylitol. In *Saccharomyces cerevisiae*, a closely related yeast species, diffusion of xylose is carried out by transport proteins encoded by hexose transporter genes HXT1, 2, 4, 5, 7 and GAL2, which all show higher affinity for hexoses such as glucose compared with pentoses [28,29]. On the other hand, it was suggested that HXT11 and HXT15 of *S. cerevisiae* are capable of transporting xylitol, although this is preceded by a long lag phase [30]. Furthermore, GXF1 (facilitated diffusion type) and GXS1 (xylose/glucose-H^+^ symport type) have been identified as xylose transporters in *Candida intermedia* [31], and CtSTP1/CtSTP2 (both facilitated diffusion type, showing a broad specificity towards a wide range of sugars) have been identified in *Candida tropicalis* [32]. Currently, the mechanisms of xylose/xylitol uptake in *C. albicans* have not been sufficiently studied, but we assume that they are similarly non-specific to xylose/xylitol, leading to lower uptake efficiency.

Another interesting and important finding was that the growth and metabolism of *C. albicans* on glucose were not inhibited at all by the presence of xylose or xylitol (Figures 1 and 2A). Furthermore, the growth and proton production patterns observed when using glucose alone, glucose and xylose or glucose and xylitol were very similar (Figures 1 and 2A). This suggests that, unlike *S. mutans*, the presence of xylose and xylitol does not lead to metabolic inhibition, including metabolically futile cycles, or even alter metabolic gene expression. These results contradict a previous report that xylitol inhibited *C. albicans* growth [33]. However, the minimum inhibitory concentration (MIC) and minimum fungicidal concentration (MFC) of xylitol in that study were 1314 and 2629 mM, respectively, which are extremely high concentrations that would be difficult to add to food without causing serious side effects. Furthermore, this inhibitory effect may be due to osmotic pressure rather than the toxicity of xylitol itself. Regarding metabolic gene expression, *Candida* species prefers glucose as an energy source and suppresses the expression of genes related to alternative carbon sources during glucose metabolism, a process known as ‘glucose repression’ [18], suggesting that the expression of enzymes related to xylose/xylitol metabolism is also suppressed in the presence of glucose.

### Proton production and metabolic end products

Proton production by *C. albicans* was highest when glucose was used as a metabolic substrate (Figure 2A), with acidic end products accounting for less than 10% of the protons produced (Figure 2B). Even more noteworthy, approximately half of the end products were non-acidic, such as ethanol and glycerol (Figure 3). Because reducing power is required for the production of ethanol and glycerol, these findings suggest that NADH obtained during glycolysis in glucose metabolism is mainly utilised for the production of ethanol and glycerol (Figure 4). In contrast, proton production during the metabolism of xylose and xylitol in *C. albicans* was low compared with growth levels (Figures 1 and 2A). This could possibly be attributed to the shorter observation time of pH-stat experiments, where measurements were recorded for only 30 min, compared to the longer time period required for growth experiments. Furthermore, as mentioned previously, the excess NADH produced during xylose and xylitol metabolism likely led to the production of non-acidic end products without CO_2_ (bicarbonate) production such as glycerol, resulting in low proton production. However, the non-acidic end products of xylose and xylitol metabolism were not analysed in the present study; therefore, further research is required.

As mentioned above, *C. albicans* showed the highest proton production from glucose regardless of the carbon source used for growth; however, among the acidic end products detected by HPLC, succinate was predominant in cells grown on glucose, whereas acetate was predominant in cells grown on xylose and xylitol (Figure 2B). These results suggest that the TCA cycle was activated during growth on glucose, whereas acetate-producing pathways were activated during growth on xylose and xylitol (Figure 4). However, this is tenuous because the organic acids detected by HPLC accounted for only a small percentage of the total end products, and the profiles of the end products were similar among cells grown on different carbon sources (Figure 3).

The acidic end products from xylose and xylitol were mainly in the form of acetate (Figure 2B). The acetate production pathway via NAD(*P*)-dependent acetaldehyde dehydrogenase (Figure 4, enzyme ⑥) may be prioritised to replenish NADPH, which is not produced in the pentose phosphate pathway during the metabolism of xylose and xylitol, as described above. However, it should be noted again that the organic acids detected by HPLC only account for a small proportion of detected protons (Figure 2B).

It is important to consider the effect of this large amount of ethanol on other members of the oral microbiome, and consequently on oral health. Ethanol produced by *Candida* species can be reconverted to acetaldehyde by streptococci, potentially increasing the risk of oral cancer [34]. Alternatively, it may inhibit the growth of ethanol-sensitive bacteria and alter the composition of the oral microbiome. In the present study, only trace amounts of acetaldehyde were detected, which may be due to the ample supply of reducing power during the fermentation process, where acetaldehyde served as the final electron acceptor [35]. It has been reported that *C. albicans* also produces acetaldehyde from ethanol [36], indicating that *C. albicans* actually possesses a metabolic pathway for producing ethanol from glucose via acetaldehyde (Figure 4).

### Overall overview of glucose metabolism based on stoichiometry

Based on the metabolic end product profile (Table 1) and previous reports [17,37–39], we estimated the overall metabolic pathway for glucose metabolism in *C. albicans* (Figure 4). The carbon recovery rate calculated from the detected metabolic end product profile was high (81.07–84.48%), and the reduction‒oxidation balance was also high (93.26–93.97%), supporting the validity of this metabolic pathway.

The carbon recovery deficit (16–19%) suggests that glucose may have accumulated intracellularly as polysaccharides [40] or other end products may have been produced. Furthermore, it is possible that some CO_2_, one of the metabolic end products, escaped from the reaction mixture. The carbon production calculated by considering that the protons exceeding the corresponding amount of organic acids detected by HPLC originated from dissolved bicarbonate ions, was lower than the CO_2_ production calculated from the predicted metabolic pathways (Table 1), supporting this possibility.

It is believed that under normoxia, *Candida* species predominantly oxidise glucose-derived pyruvate using the TCA cycle and electron transport chain, producing CO_2_ and water without organic acids and non-acidic end products [41]. However, our results suggest that under aerobic conditions with abundant carbohydrates, such as those used in the present experiment, *C. albicans* may metabolise carbohydrates primarily through glycolysis and part of the reverse TCA cycle (Figure 4, phosphoenolpyruvate → oxaloacetate → malate → fumarate → succinate), and produce organic acids, CO_2_ and non-acidic end products such as ethanol and glycerol by consuming the reducing power generated during the metabolic process (Figure 4). It was reported that even under aerobic conditions, when high concentrations of glucose were present, certain yeasts prioritised fermentation, which produced ethanol and CO_2_ from glucose via glycolysis, and suppressed energy-efficient respiration [35,42]. This phenomenon is known as the ‘Crabtree effect’ [42]. Crabtree-positive yeasts are suggested to sacrifice efficient ATP production to prioritise rapid glucose consumption in order to starve competing micro-organisms, producing ethanol as a preservative that inhibits their growth [35]. Historically, *C. albicans* has been considered a Crabtree-negative yeast whose respiration is unaffected by the presence of glucose [43]. However, increasing evidence suggests that glucose actually inhibits the respiration of *C. albicans*, which shares some characteristics of Crabtree-positive yeasts [44,45]. This is similar to our results showing that *C. albicans* produced large amounts of ethanol from glucose even under aerobic conditions.

## Conclusion

*C. albicans* metabolised xylose and xylitol under aerobic conditions and produced acid. Unlike *S. mutans*, neither xylose nor xylitol inhibited its glucose metabolism. However, the level of acid production from xylose and xylitol was low, suggesting their low cariogenic potential in the oral environment. It is important to note that these results were obtained using only the type strain of *C. albicans*, mainly in yeast form; varying clinical isolates, or cells in the hyphal forms may exhibit different behaviours due to metabolic changes, requiring further investigation. This study revealed that *C. albicans* ferments sugars, producing large amounts of ethanol and CO_2_ along with organic acids. The overall picture of oral *Candida* sugar metabolism obtained for the first time in the present study provides new information when considering the metabolic function of *Candida* species within the oral microbiome.

## Supplementary Material

Supplementary material Supplemental Figure

Supplementary material Figure s1

Supplementary material Figure S2
