# Supplementary material for: Characteristics of Candida albicans metabolism of glucose and two sugar substitutes, xylose and xylitol and effect of these substitutes on glucose metabolism from a cariogenic perspective
Source: J Oral Microbiol. 2026 Feb 7;18(1):2626130. doi: 10.1080/20002297.2026.2626130 (PMC12884996; doi:10.1080/20002297.2026.2626130)
Supplement: Supplementary material — Supplemental Figure [file ZJOM_A_2626130_SM2896.docx]

**Supplemental Figure 1.** Representative high performance liquid chromatography (HPLC) chromatograms of the sample and standard solutions.

**Supplemental Figure 2.** Growth (OD_520_) of *C. albicans* JCM1537, JCM2903, and JCM2085 using glucose, xylose, or xylitol as a substrate under aerobic conditions. Data are shown as the mean ± standard deviation (SD) of 3 independent experiments.
