# Supplementary figures and images for: Characteristics of Candida albicans metabolism of glucose and two sugar substitutes, xylose and xylitol and effect of these substitutes on glucose metabolism from a cariogenic perspective
Source: J Oral Microbiol. 2026 Feb 7;18(1):2626130. doi: 10.1080/20002297.2026.2626130 (PMC12884996; doi:10.1080/20002297.2026.2626130)

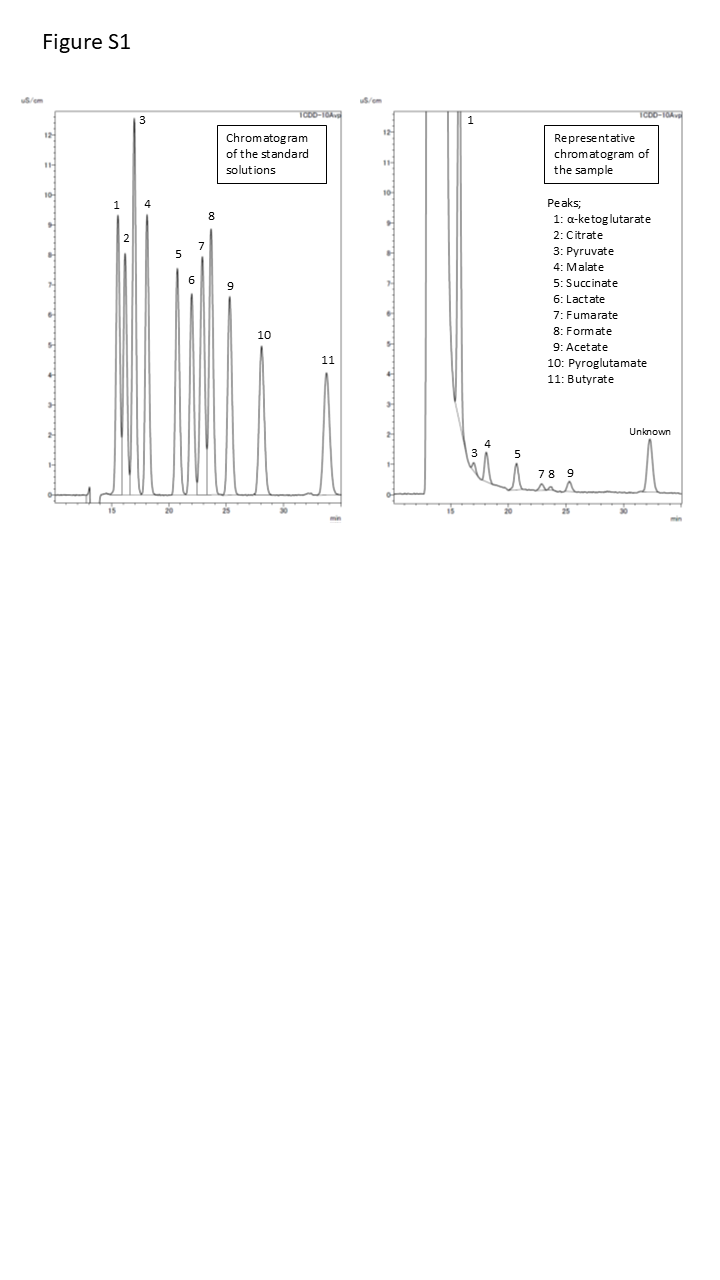

Supplement: Supplementary material — Figure s1 [file ZJOM_A_2626130_SM2894.tif]

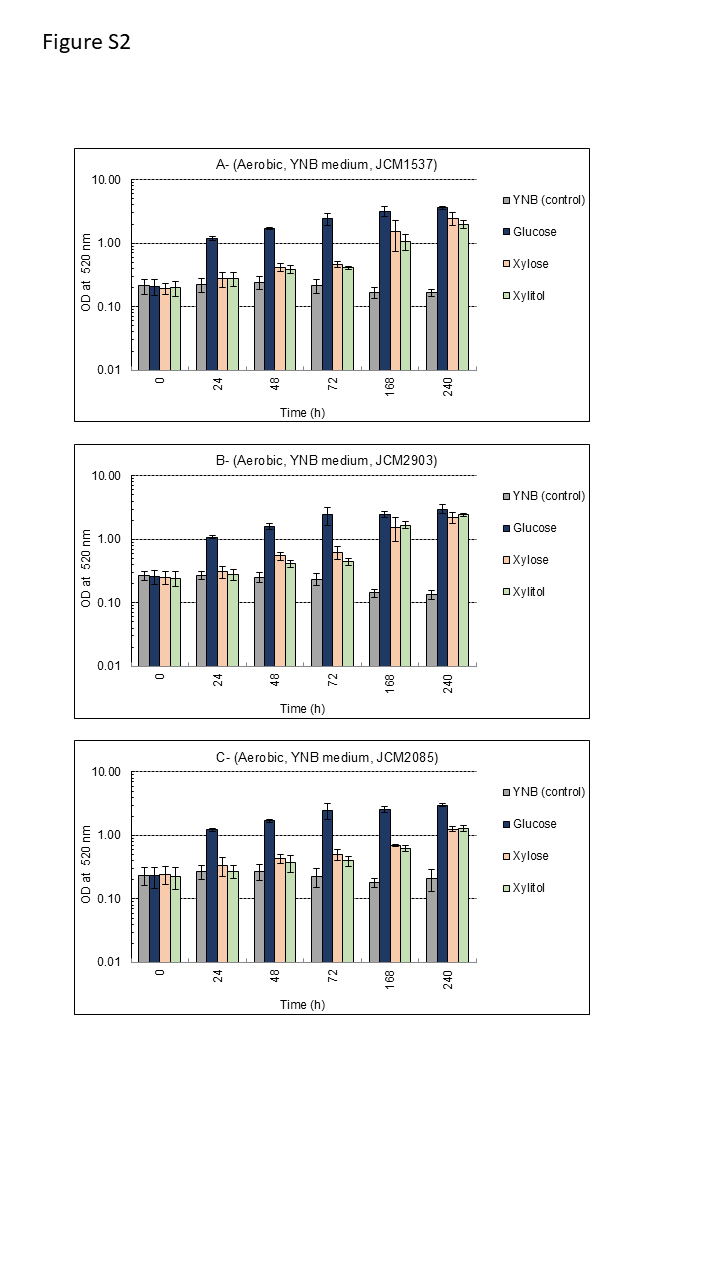

Supplement: Supplementary material — Figure S2 [file ZJOM_A_2626130_SM2895.tif]
